# Gender Prejudice Within the Family: The Relation Between Parents' Sexism and Their Socialization Values

**DOI:** 10.3389/fpsyg.2022.846016

**Published:** 2022-02-24

**Authors:** Daniela Barni, Caterina Fiorilli, Luciano Romano, Ioana Zagrean, Sara Alfieri, Claudia Russo

**Affiliations:** ^1^Department of Human and Social Sciences, Università degli Studi di Bergamo, Bergamo, Italy; ^2^Department of Human Sciences, Libera Università Maria SS. Assunta (LUMSA), Rome, Italy; ^3^Department of Human Sciences, Universitá Europea di Roma (UER), Rome, Italy; ^4^Department of Psychology, Università Cattolica del Sacro Cuore di Milano, Milan, Italy

**Keywords:** gender prejudice, hostile sexism, benevolent sexism, parents, socialization values

## Abstract

Gender inequalities are still persistent despite the growing policy efforts to combat them. Sexism, which is an evaluative tendency leading to different treatment of people based on their sex and to denigration (hostile sexism) or enhancement (benevolent sexism) of certain dispositions as gendered attributes, plays a significant role in strengthening these social inequalities. As it happens with many other attitudes, sexism is mainly transmitted by influencing parental styles and socialization practices. This study focused on the association between parents' hostile and benevolent sexism toward women and their socialization values (specifically, conservation and self-transcendence), that are the values parents would like their children to endorse. We took both parents' and children's sex into account in the analyses. One-hundred-sixty-five Italian parental couples with young adult children participated in the study. Parents, both the mother and the father, individually filled in a self-report questionnaire composed of the Ambivalent Sexism Inventory and the Portrait Values Questionnaire. Findings showed that mothers' benevolent sexism was positively related to their desire to transmit conservation values to their sons and daughters. This result was also found for fathers, but with a moderation effect of children's sex. Indeed, the positive relationship between fathers' benevolent sexism and conservation was stronger in the case of sons than of daughters. Moreover, fathers' benevolent sexism was positively associated with self-transcendence values. Finally, fathers' hostile sexism was positively associated with conservation and negatively with self-transcendence. Limitations of the study, future research developments, and practical implications of the results are discussed.

## Introduction

Despite growing policy efforts designed to foster gender equality, culturally rooted and persistent inequalities are still around, and gender prejudice and sexism are thought to contribute significantly to this (Vandenbossche et al., 2018). Generally speaking, sexism is a form of prejudice and discrimination based on stereotypical beliefs about sex or gender (Dovidio et al., 2008). In their Ambivalent Sexism Theory, Glick and Fiske (1996) innovatively considered sexism as a multidimensional construct composed of two sets of sexist attitudes, namely hostile and benevolent sexism. Both the forms of sexism fuel the subordination of women to men, although they deeply differ in their expression (Mastari et al., 2019). Hostile sexism refers to the traditional conceptualization of sexism as a reflection of hostility against women. Men are perceived as dominant over women, and the women who do not respect the conventional gender roles represent a potential threat to social order and men's power. Benevolent sexism, instead, is expressed in a seemingly positive and more subtle way. Women are paternalistically seen as loving but fragile individuals and therefore need men's protection and support. This protection is granted in exchange for women's respect of traditional gender roles (Glick and Fiske, 2001).

From childhood to young adulthood, parents play a key role in their children's development of gender-role attitudes and stereotypes (e.g., Halpern and Perry-Jenkins, 2016). Nevertheless, only a few empirical studies deal with hostile and benevolent sexism within the family. In general, the higher is parents' sexism, the stronger are their expectations that children behave in line with gender stereotypes. As a matter of fact, Garaigordobil and Aliri (2011) found a direct relationship between parents' hostile and benevolent sexist attitudes and their adolescent children's sexist attitudes, thus suggesting an intergenerational transmission of them (see also Montañés et al., 2012). The strength of this connection varied according to parents' and adolescents' sex, being higher between the mothers' and daughters' sexism and between the fathers' and sons' sexism. Effectively, parenting practices tend to reinforce gender-typed behaviors mainly, but not exclusively, within the same-sex parent-child dyads (e.g., Grusec and Goodnow, 1994; Lund et al., 2002). Lipowska et al. (2016), in their research concerning parental attitudes of couples with young children, showed the association between parents' sexism and parenting styles. The authors reported that fathers' sexism (both hostile and benevolent) was positively associated with inconsequence attitudes (i.e., unpredictable parenting behavior, mainly depending on parents' current mood) toward sons. In the case of daughters, fathers' hostile sexism supported overprotective attitudes, while the benevolent one was positively related to promoting autonomy. On the other side, mothers' benevolent sexism was negatively associated with overprotective and demanding attitudes toward sons, but not toward daughters. From Garaigordobil and Aliri (2012), which involved parental couples of adolescents, it turned out that parents' indulgent style (i.e., high involvement and low imposition) had the strongest relationship with a low level of adolescents' sexism (regardless of adolescents' sex).

Parents' socialization values are at the core of parenting styles and practices (e.g., Grusec and Goodnow, 1994; Kikas et al., 2014). Socialization values are the values parents would like their children to endorse (Barni et al., 2017), and they guide parents in raising and socializing their children both in the short-term (i.e., what values parents pursue for their children in the present) and in a long-term perspective (i.e., what values parents would like to see in their children in adulthood) (Lasker and Lasker, 1991; Tulviste et al., 2012). Previous studies mainly relied on Schwartz's Theory of basic human values (Schwartz, 1992, 2012) and showed that parents (both mothers and fathers) would like their sons and daughters to give importance to conservation values (i.e., tradition, conformity, and security) and self-transcendence values (i.e., benevolence and universalism) (Ranieri and Barni, 2012; Barni et al., 2017). Conservation and self-transcendence are both conceptualized as social-focused values because they mainly regulate the way people are socially related to others, relying on a principle of cooperation. However, they significantly differ from each other. On the one side, conservation values are self-protective values because they comply with the need to avoid conflicts, unpredictability, and changes. On the other side, self-transcendence values adhere to the need for relatedness, emphasizing the concern for the welfare of others and underlying self-expansive motivations (Schwartz, 2012; Russo et al., 2021).

### The Present Study

Despite the relevance of both parents' sexism and socialization values in children's education and development, to the best of our knowledge, until now no studies have examined the association between them. This study aims to overcome this gap by analyzing the moderation effect of child's sex on the relation between parental sexism (i.e., hostile and benevolent sexism toward women) and the social-focused values (i.e., conservation and self-transcendence) parents would like to transmit to their young adult children.

The study involved Italian mothers and fathers. Italy is far from reaching satisfactory results in gender equality, despite relevant progress under the pressure of women's rights movements, civil society, and local and European legislation (Rosselli, 2014). In Italy, more and more young adults live with their parents for a long time. Young adulthood is an understudied stage of life concerning sexist socialization experiences, even though an increasing number of psychological studies have reported the important role of sexism in young romantic couples' birth, dynamics, and wellbeing (Lachance-Grzela et al., 2021).

We expected to find significant associations between parents' sexism and socialization values. In particular, we hypothesized that both hostile and benevolent sexism was positively associated with conservation values, which emphasize the importance of traditions and preservation of the status quo (Schwartz, 1992). On the contrary, we could hypothesize a negative relation between hostile sexism and self-transcendence values, emphasizing the importance of benevolence, gender equality, and social justice. It is, instead, not possible to make a sound hypothesis about the relation between benevolent sexism and self-transcendence. This is because, on the one side, benevolent sexism contributes to gender inequality and, on the other side, it promotes helping behaviors and intimate relationships. Moreover, given the absence of previous research on the topic, we did not formulate any specific hypotheses about the influence of parents' and children's sex on these associations.

## Method

### Participants and Procedure

One-hundred-sixty-five Italian married couples (mothers: M_age_ = 50.85, SD = 4.51; fathers: M_age_ = 53.98, SD = 5.47) with at least one young adult (M_age_ = 22.87, SD = 2.32) son (34.8%) or daughter (65.2%)^1^ participated in the study, for a total of 330 participants. The couples were married for an average of 26.96 years (SD = 4.98) and lived in the North of Italy.

Parents were recruited through the collaboration of the universities attended by their young adult children. After being informed about the study nature and participants' rights, the parents who agreed to participate received two versions of an anonymous self-report questionnaire, one for the mother and one for the father. They completed them at home with the opportunity to phone researchers if any help was needed.

### Measures

#### Sexism

The Ambivalent Sexism Inventory (Glick and Fiske, 1996, Italian adaptation by Manganelli Rattazzi et al., 2008) was used to measure parents' sexist attitudes. The scale is composed of 22 items on a 6-points Likert scale (0 = “Completely disagree”; 5 = “Completely agree”) set into hostile sexism (item examples: “Women get offended too easily,” “Most women fail to appreciate fully all that men do for them”; α_mother_ = 0.86; α_father_= 0.88) and benevolent sexism (item examples: “Women have a superior moral sensibility,” “Women should be cherished and protected by men”; α_mother_ = 0.81; α_father_ = 0.78).

#### Socialization Values

The subscales of conservation and self-transcendence values were extracted from the Portrait Values Questionaire (Schwartz et al., 2001) and adapted to measure parents' socialization values (Barni et al., 2017). Conservation includes 13 verbal portraits describing a person's goals, aspirations, or wishes that implicitly point to the importance of a value (item example: “She/he believes that people should do what they are told. She/he is convinced that people should always follow the rules, even when no one is checking”; α_mother_ = 0.85; α_father_ = 0.84). Self-transcendence includes 10 verbal portraits (item example: “It is very important for her/him to help the people around her/him. She/he aspires to take care of their wellbeing”; α_mother_ = 0.84; α_father_ = 0.86). Parents were asked to indicate their responses to the question: “How would you want your child to respond to each item?” on a 6-points Likert scale (1 = “Not like her/him” at all; 6 = “Very much like her/him”).

### Data Analysis

Preliminarily, we performed descriptive statistics of the study's variables and correlations between them. Then, we estimated four multiple hierarchical regression models to test the moderation effect of child's sex on the relations between mothers' and fathers' sexist attitudes and their socialization values. In the first two regression models, the outcome variables were mothers' conservation and self-transcendence, respectively. In the third and fourth models, the outcome variables were fathers' conservation and self-transcendence, respectively. In all the models, the independent variables were: children's age (Step 1), children's sex (1 = sons, 2 = daughters), parents' benevolent and hostile sexism (Step 2), and the interaction terms between parents' sexism and children's sex (Step 3). Before calculating the interaction terms, the single scores of continuous variables were centered on their means to reduce the risk of collinearity (Aiken and West, 1991).

The analyses were run using SPSS v.21.0 (George and Mallery, 2013) and Interaction! (Soper, 2010).

## Results

In Table 1 descriptive statistics and correlations between the study's variables are reported.

**Table 1:** Descriptive statistics and correlations.

	**M**	**SD**	**Min**	**Max**	**SK**	**K**	**3**	**4**	**5**	**6**	**7**	**8**	**9**	**10**
1. CSEX	–	–	–	–	–	–	0.03	0.05	0.16^*^	−0.01	−0.03	0.05	−0.03	0.01
2. CAGE	22.87	2.32	20	31	0.95	0.86	−0.08	−0.17^*^	−0.03	−0.11	0.03	0.04	0.12	0.04
3. MHOSTSEX	2.01	0.92	0	4.10	−0.05	−0.54		0.64^**^	0.33^**^	0.17^*^	0.28^**^	0.09	−0.15	−0.18^*^
4. MBENSEX	2.32	0.90	0	4.60	−0.35	−0.14			0.27^**^	0.20^*^	0.34^**^	0.18^*^	−0.17^*^	−0.12
5. FHOSTSEX	2.50	0.95	0.10	5.00	−0.12	−0.31				0.27^**^	0.16^*^	0.23^**^	−0.14	−0.25^**^
6. FBENSEX	2.84	0.82	0.70	4.60	−0.38	−0.20					0.07	0.23^**^	0.03	0.10
7. MCONS	4.00	0.79	1.92	6.00	−0.27	−0.08						0.19^*^	0.38^**^	−0.08
8. FCONS	4.16	0.71	2.15	5.85	−0.10	−0.16							0.04	0.48^**^
9. MSELFT	4.90	0.60	3.10	6.00	−0.48	0.05								0.18^*^
10. FSELFT	4.81	0.68	2.80	6.00	−0.43	0.04								

* *p < 0.05*,

** *p < 0.01 (2-tails)*;

*M, Mean; SD, Standard deviation; SK, Skewness; K, Kurtosis; CSEX, Children's sex; CAGE, Children's age; MHOSTSEX, Mothers' hostile sexism; MBENSEX, Mothers' benevolent sexism; FHOSTSEX, Fathers' hostile sexism; FBENSEX, Fathers' benevolent sexism; MCONS, Mothers' conservation; FCONS, Fathers' conservation; MSELFT, Mothers' self-transcendence; FSELFT, Fathers' self-transcendence*.

Table 2 shows the results of the two hierarchical regression models referred to mothers' variables.

**Table 2:** Hierarchical multiple regression models with mothers' variables.

	**Conservation values**			**Self-transcendence values**		
	**β**	***t***	**Model summary**	**β**	***t***	**Model summary**
Step 1			*R*^2^ = 0.00			*R*^2^ = 0.02
CAGE	0.03	0.36	*F*_(1, 154)_ = 0.13	0.12	1.53	*F*_(1, 154)_ = 2.34
Step 2			*R*^2^ = 0.12^**^			*R*^2^ = 0.04
CSEX	−0.04	−0.51	*F*_(4, 151)_ = 5.40	0.00	0.00	*F*_(4, 151)_ = 1.59
MHOSTSEX	0.07	0.65	Δ*R*^2^ = 0.12	−0.07	−0.66	Δ*R*^2^ = 0.02
MBENSEX	0.31	3.04^*^		−0.11	−1.00	
Step 3			*R*^2^ = 0.13^*^			*R*^2^ = 0.04
MHOSTSEX*CSEX	0.45	1.10	*F*_(6, 149)_ = 3.84	−0.18	−0.41	*F*_(6, 149)_ = 1.12
MBENSEX*CSEX	−0.50	−1.15	Δ*R*^2^ = 0.01	0.29	0.63	Δ*R*^2^ = 0.00

* *p < 0.05*,

** *p < 0.01*,

*R^2^ = R-square; ΔR^2^ = R-square changes; CAGE, Children's age; CSEX, Children's sex; MHOSTSEX, Mothers' hostile sexism; MBENSEX, Mothers' benevolent sexism*.

Only the association between mothers' benevolent sexism and conservation was statistically significant: the more mothers endorsed benevolent sexism, the more they wanted their sons and daughters to give importance to values such as tradition, conformity, and security. Children's sex did not moderate any associations between mothers' sexism and socialization values.

Table 3 contains the results of the two hierarchical regression models referred to fathers' variables.

**Table 3:** Hierarchical multiple regression models with fathers' variables.

	**Conservation values**			**Self-transcendence values**		
	**β**	***t***	**Model summary**	**β**	***t***	**Model summary**
Step 1			*R*^2^ = 0.00			*R*^2^ = 0.00
CAGE	0.04	0.52	*F*_(1, 154)_ = 0.27	0.04	0.51	*F*_(1, 154)_ = 0.26
Step 2			*R*^2^ = 0.09^**^			*R*^2^ = 0.11^**^
CSEX	0.03	0.34	*F*_(4, 151)_ = 3.59	0.07	0.93	*F*_(4, 151)_ = 4.73
FHOSTSEX	0.16	2.00^*^	Δ*R*^2^ = 0.09	−0.32	−3.98^**^	Δ*R*^2^ = 0.11
FBENSEX	0.20	2.47^*^		0.21	2.65^**^	
Step 3			*R*^2^ = 0.13^**^			*R*^2^ = 0.13^**^
FHOSTSEX*CSEX	0.45	1.55	*F*_(6, 149)_ = 3.47	0.47	1.62	*F*_(6, 149)_ = 3.62
FBENSEX*CSEX	−0.65	−2.18^*^	Δ*R*^2^ = 0.04	−0.17	−0.59	Δ*R*^2^ = 0.02

* *p < 0.05*,

** *p < 0.01*,

*R^2^ = R-square; ΔR^2^ = R-square changes; CAGE, Children's age; CSEX, Children's sex; FHOSTSEX, Fathers' hostile sexism; FBENSEX, Fathers' benevolent sexism*.

**Figure 1:**
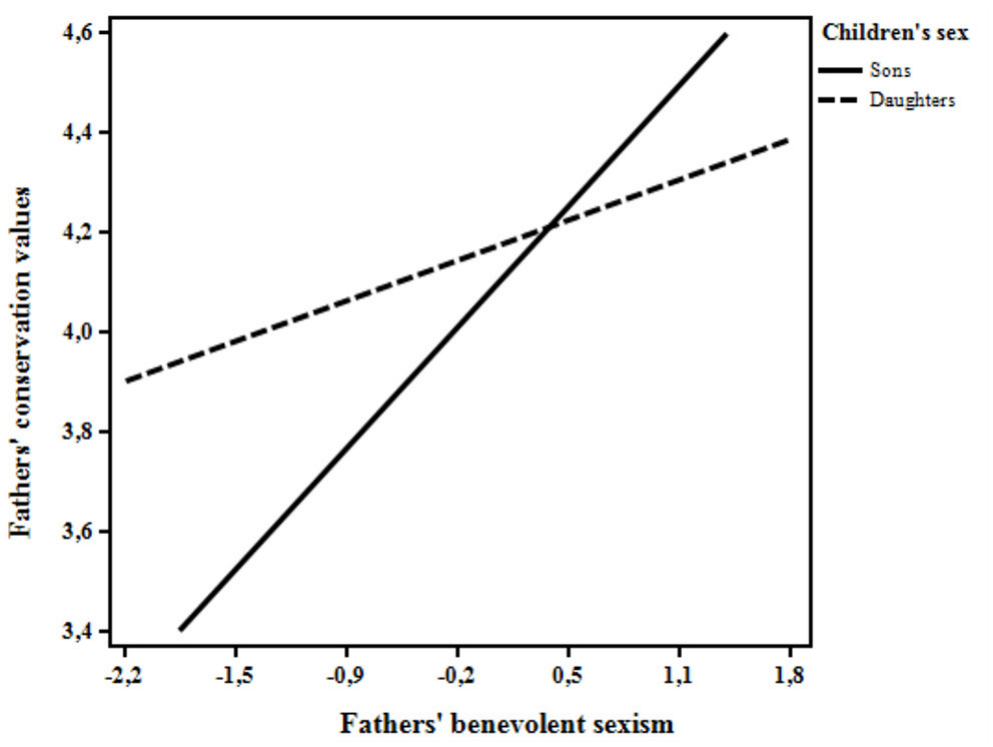
The moderating role of children's sex on the relation between fathers' benevolent sexism and fathers' conservation values.

Findings showed that fathers' hostile and benevolent sexist attitudes were positively related to conservation. Besides, fathers' hostile and benevolent attitudes were significantly related to self-transcendence, but in opposite directions (negative for hostile sexism and positive for benevolent sexism). Interestingly, children's sex moderated the relation between fathers' benevolent sexism and conservation values. As illustrated in Figure 1, the simple slope analysis revealed that the positive link between benevolent sexism and conservation was stronger in the case of sons [Simple slope = 0.37, SE = 0.12; 95% CI (0.13, 0.61), *p* < 0.01], than in the case of daughters [Simple slope = 0.12, SE = 0.07; 95% CI (−0.02, 0.27), *p* > 0.05].

## Discussion

The current study explored the association between parents' hostile and benevolent sexism toward women and socialization values. In particular, we considered the social-focused values (i.e., conservation and self-transcendence), which contribute to regulating how people relate socially to each other (Schwartz, 2012). We involved both mothers and fathers and analyzed the moderation effect of children's sex on the sexism-socialization values link.

The straightforward result is that parents' sexism is significantly associated with the social-focused values parents would like to see in their children. There are two related points to this main result: first, parents' benevolent sexism, more than the hostile one, seems to be involved in children's value socialization; second, fathers' sexism, more than the mothers' one, intervenes in children's value socialization.

As hypothesized, mothers' and fathers' benevolent sexism was positively related to conservation values. Parents characterized by high levels of benevolent sexism might want to pass on to their children conservation values because the content of these values is in line with the desire for a stable context in which women are protected from harm (Sortheix and Schwartz, 2017). It is worthwhile noting that the relationship between benevolent sexism and conservation to transmit to sons and daughters is the only one significant for mothers. Benevolent sexism is the most subtle type of sexism, generally endorsed by both genders, which includes valuing feminine-stereotypes (Mastari et al., 2019). Conservation values are self-protective values, that serve to cope with anxiety due to uncertainty in the world by avoinding conflict (conformity) and maintaining the current order (tradition and security). Thus, the relation between mothers' benevolent sexism and the socialization values of conservation may express the desire to ensure a long-term safety for women (across generations) by the fulfillment of traditional gender roles.

Interestingly, as shown by the moderated regression analysis, the positive relation between fathers' benevolent sexism and conservation was moderated by children's sex, being stronger in the case of sons than of daughters. Thus, from the fathers' view, it is the task of men (i.e., sons) to preserve stability and safety in order to protect and support women. Furthermore, fathers' benevolent sexism was related to growth and self-expansive values (i.e., self-transcendence). Fathers with high levels of benevolent sexism would likely interpret their sexist attitude as a form of respect and care toward women instead of an attitude hindering women's freedom. Their sexism might assume the shape of paternalism, thus strengthening their view of being a caring person (Glick and Fiske, 2001). Hence, they may wish to transmit to their children generative values (Erickson, 1963) whose content is related to the concern for the welfare and the protection of all human beings (Schwartz, 2012).

On the contrary, fathers' hostile sexism was not generative at all. It was positively associated with conservation values, but negatively with self-transcendence values. Men with high levels of hostile sexism tend to exhibit a hostile attitude toward women, reinforcing the view that women are only suited for domestic roles, even when women aspire to high-status roles that are perceived as suitable only for men (Eagly and Mladinic, 1994). As such, fathers' hostile sexism discourages the promotion of self-transcendence values that emphasize the understanding, appreciation, tolerance, and equality of all people.

Two main limitations of the present study must be acknowledged. First, the study's cross-sectional design did not allow us to draw causal interpretations from the results or catch potential changes over time. Second, the sample was of convenience and relatively small in size, with fewer sons than daughters. For these reasons, future longitudinal studies with larger representative sample of families are needed to better understand the role of parents' sexism within the family socialization processes.

Despite its limitations, this is the first study showing that parents' sexism intervenes at the core of socialization of young adult children by being related to what parents would like their children to value. There is a direct transmission of sexist attitudes between parents and children (Garaigordobil and Aliri, 2011) and an indirect path through promoting desired values across generations. Values represent, to some extent, a family heritage (e.g., Fiorilli et al., 2015). Parents have a mental representation of an “ideal adult,” developed based on their own values, beliefs, and (sexist) attitudes that shape what they consider beneficial and adaptive. When they state the desired values for their children, they project such representation onto them (Rosenthal and Roer-Strier, 2006; Barni et al., 2017).

All in all, our results highlighted the “pervasive” role of fathers' sexism in children's value socialization. These results align with previous research showing the stronger influence of fathers' hostile and benevolent sexism on family relationships and dynamics (e.g., aggressive parenting, Overall et al., 2021). In the socialization of sexism, this seems especially true for the father-son dyad as suggested by our and previous studies (e.g., Garaigordobil and Aliri, 2011).

This study's findings can have significant practical implications. Interventions to reduce sexism are quite rare, practically absent in working with parents. Differently from other forms of prejudice (e.g., racial), intergroup contact cannot be applied to reduce sex prejudice. Providing individuals with gender-relevant information could be a good starting point to change their sexist attitudes (Becker and Swim, 2011). In this line, it would be helpful to develop training programs involving both mothers and fathers to strengthen parents' awareness about their hostile and benevolent sexist attitudes in order to avoid directly or indirectly transmitting them to future generations. We must not forget that cultural persistence is essentially the result of family and social transmission (Schönpflug and Bilz, 2009).

## Data Availability Statement

The raw data supporting the conclusions of this article will be made available by the authors, without undue reservation.

## Ethics Statement

The study was reviewed and approved by the Scientific Board of the Family Studies and Research University Centre, Catholic University of Milan, and by the Ethical Committee of the Catholic University of Milan, Department of Psychology, Italy. The authors assert that all procedures contributing to this work comply with the ethical standards of the relevant national and international committees on human experimentation. All participants gave their written informed consent to participate in this study.

## Author Contributions

DB designed the study, collected the data, and contributed to the writing of the manuscript. SA designed the study, collected the data, and edited the manuscript. LR and IZ analyzed the data and wrote the Methods and Results. CF and CR contributed to the writing of Introduction and Discussion. All authors contributed to the article and approved the submitted version.

## Funding

This work was supported by research funds granted to the DB by the University of Bergamo.

## Conflict of Interest

The authors declare that the research was conducted in the absence of any commercial or financial relationships that could be construed as a potential conflict of interest.

## Publisher's Note

All claims expressed in this article are solely those of the authors and do not necessarily represent those of their affiliated organizations, or those of the publisher, the editors and the reviewers. Any product that may be evaluated in this article, or claim that may be made by its manufacturer, is not guaranteed or endorsed by the publisher.

## Footnotes

^1^If parents had more than one young-adult child, they were asked to respond thinking about their firstborn.
